# Toward Age-Friendly High Education: An Intergenerational Participatory Co-Design Approach

**DOI:** 10.1093/geroni/igab046.2017

**Published:** 2021-12-17

**Authors:** W Q Lou Vivian, Esther Woo, Nicol Pan, Peter J Cobb, Xiao Hu, Michael Cheng

**Affiliations:** 1 The University of Hong Kong, Hong Kong, Hong Kong; 2 The University of Hong Kong, The University of Hong Kong, Hong Kong

## Abstract

Objective: When aging becomes a global challenging, we believe it is timely important to equip aging knowledge among university students regardless of their disciplinary study subjects. This study aims to describe principles and process of development an aging-related curriculum in high education entitled “Intergenerational Participatory Co-design Project (IPCP)” and evaluate its impacts. Methodology: Guided by a key principle of involving participants of any learning context as co-creators of both the learning process and learning outcomes, IPCP went through four stages of development including capacity building, co-creation on learning objectives, deliberated content learning, and learning outcome dissemination. Mixed methodology including qualitative in-depth interview and quantitative questionnaire were applied in evaluation. A total of 26 participants, from three generations recruited from one university, one secondary school, and a pool of senior champions under a geron-infusion initiative participated. Findings: after attaining capacity building workshops applying Optimal Quality Intergeneration Interaction Framework, three learning groups formulated. A common theme “preserving cultural heritage” emerged, while each group has identified a specified focus (e.g., food, Tai Ji, and historic sites guide). Quotes collected and survey data revealed positive impacts in reducing stereotype and enhancing learning experiences. Conclusion: IPCP demonstrated good practices in role models in multi-disciplinary collaboration in pedagogy innovation. It also paved solid way towards a learning community interwoven with continuous innovation: IPCP becomes a pioneer contributor of library’s digital data hub solution; common core office starts to develop a human lifespan cluster; two research team members started new collaboration on geron-infusion in Faculty of Education.

